# Radiofrequency‐transparent local B_0_ shimming coils using float traps

**DOI:** 10.1002/mrm.30361

**Published:** 2024-11-04

**Authors:** Changzhe Liu, Hao Liang, Ming Lu, John C. Gore, Saikat Sengupta, Xinqiang Yan

**Affiliations:** ^1^ Department of Electrical and Computer Engineering Vanderbilt University Nashville Tennessee USA; ^2^ Vanderbilt University Institute of Imaging Science Vanderbilt University Medical Center Nashville Tennessee USA; ^3^ Department of Radiology and Radiological Sciences Vanderbilt University Medical Center Nashville Tennessee USA

**Keywords:** B_0_ shimming, implant, multicoil shimming, RF transparency, signal‐to‐noise ratio (SNR), transmit field

## Abstract

**Purpose:**

Static field (B_0_) inhomogeneities present a major challenge in high‐field MRI. Multicoil shimming using independent, local, direct‐current (DC) shim coils has emerged as a powerful and flexible technique to address this issue. However, many‐turn DC coils can lead to significant mutual coupling with radiofrequency (RF) coils, causing transmit field (B_1_^+^) distortions and signal‐to‐noise ratio degradation.

**Methods:**

We introduce an innovative RF‐transparent DC coil that performs B_0_ shimming while minimizing RF performance impact. The design incorporates float traps to maintain high RF impedance, allowing flexible placement relative to the RF coil without compromising signal‐to‐noise ratio or affecting B_1_^+^. We fabricated square‐shaped DC coils with float traps for 3T MRI and compared them with conventional DC coils. To demonstrate high ΔB_0_/Amp efficiency, we conducted a B_0_ shimming experiment around a metal hip implant.

**Results:**

Bench tests and MRI experimental results demonstrated that the RF‐transparent DC coil effectively minimized RF interference, preserved signal‐to‐noise ratio, and maintained B_1_^+^, even when placed near the RF receive coil. Additionally, the DC coil significantly improved B_0_ homogeneity near metal implants and substantially reduced image distortion.

**Conclusion:**

The RF‐transparent DC coil offers a flexible, effective solution for managing B_0_ inhomogeneities, paving the way for integrating multiturn DC coils in clinical MRI settings without extensive hardware modifications.

## 1 INTRODUCTION

Static field (B_0_) inhomogeneities are a persistent and significant challenge in MRI and MR spectroscopy, as they can lead to image artifacts, signal losses, spectral and image distortions, and line broadening, particularly in high‐field systems.^1^, ^2^, ^3^ Traditionally, sample B_0_ inhomogeneities have been addressed by the use of in‐bore shim coils, each of which contributes field perturbations corresponding to spherical harmonics of different orders.^4^, ^5^ Recently, multicoil shimming using independent local direct‐current (DC) shim coils has emerged as a powerful and flexible ancillary technique to address this problem.^6^, ^7^, ^8^, ^9^

Two main approaches to DC shimming have been developed. One uses separate DC shim coils,^10^ while the other integrates the DC shim with the radiofrequency (RF) coil.^8^, ^9^ Combined RF/DC coils using shared conductors have advantages in saving valuable space and avoiding crosstalk between the DC and RF components. However, combined RF/DC coils have other limitations. First, the size, shape, and number of DC coils are determined by the RF coil, owing to the higher priority of selecting RF coils that provide optimal signal‐to‐noise ratio (SNR). Second, the number of turns in the DC coil is limited to one or two, due to limitations on the intrinsic design of RF coils at high fields. Third, RF/DC coils are generally customized and hard to implement in existing, in‐use, commercial RF coils.

Separate local DC shimming coils allow for multiple turns and greater freedom in geometric and spatial configuration. However, placing many‐turn DC coils near or inside the RF coil can lead to significant mutual coupling between the RF and DC coils, resulting in transmit field (B_1_^+^) distortions and SNR degradation. In Juchem et al.,^10^ the DC coils were shifted out of the longitudinal coverage of a local transmit/receive RF array. However, this approach restricts the placement of local shim coils and still has coupling problems if the body coil is used for RF transmission. In Zhou et al.,^11^ the DC coils were placed orthogonal to the RF receive coils to mitigate their coupling. However, this design is constrained by the space inside the bore and is limited in the number, geometry, and efficiency of the DC coil setup. Another method to achieve isolation is to insert an LC tank circuit or a large RF choke in line with the DC coil.^12^, ^13^ Although this is feasible for coils with a few turns, it becomes challenging for a larger number of turns and may not effectively decouple the DC coil from the RF coil.

This report introduces a novel, RF‐transparent DC coil designed to shape the B_0_ field with minimal impact on RF performance. This coil design uses a float trap to create high RF impedance for every turn of the DC coil that passes through it. This design aims to be “transparent” to the RF field, allowing for free placement relative to the RF coil without compromising either the B_1_^+^ field or SNR. A preliminary description of this work was reported in Yan.^14^

## 2 METHODS

### 2.1 Concept, hardware fabrication, and bench test

When DC coils have multiple turns, the large inductance and parasitic capacitance may impair the RF field even though the self‐resonant frequency is still far from the RF frequency. The parasitic parameters in many‐turn coils are often unpredictable and can lead to significant coupling issues with Rx coils.

**FIGURE 1:**
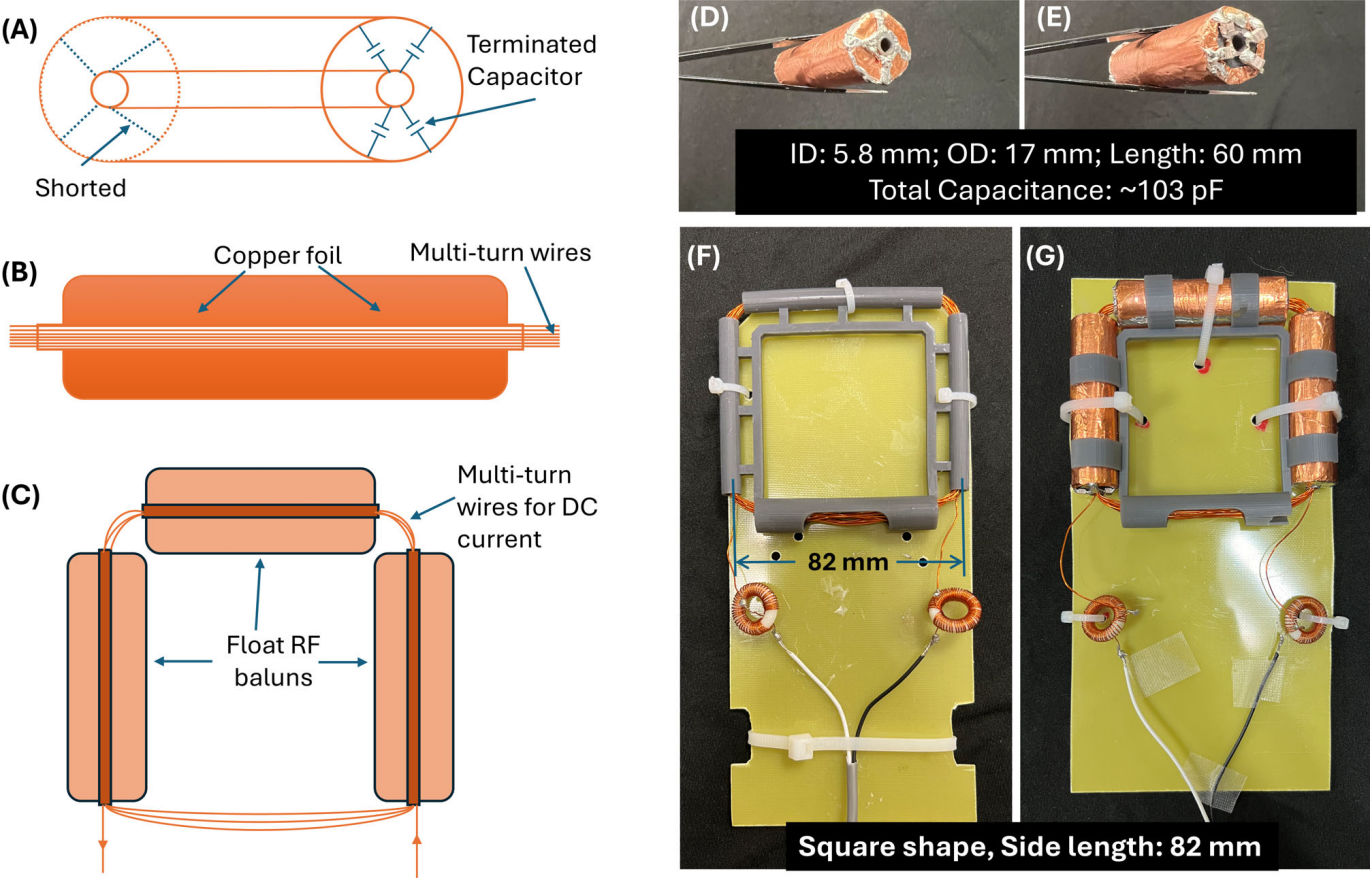
Design and construction of the transparent direct‐current (DC) coil. (A) Schematic diagram of the terminated capacitor configuration, showing the shorted and terminated capacitors of the balun. This figure demonstrates a float balun with the terminated capacitors positioned at one end. Note that they could be terminated at both ends or in the middle. (B) Cross‐sectional view of the balun illustrating the placement of copper foil and multi‐turn wires. (C) Schematic of the complete coil design, including multiturn wires for DC current and float radiofrequency (RF) baluns. (D) Photograph of a single float RF balun. (E) Another view of the float RF balun demonstrating its capacitor soldering. (F) Top view of the normal DC coil in a square shape, showing the coil configuration and copper wire layout. (G) Top view of the transparent DC coil, highlighting the arrangement of the float RF baluns and multiturn wires. ID, inner diameter; OD, outer diameter.

A key point of our design is the float trap, proposed by Seeber et al.^15^ to reduce the common‐mode current along the shield of coaxial cables, as shown in Figure 1A. The float trap is essentially a cylindrical resonator that blocks RF signals passing through it.

We attempted such a design for 3 T by fabricating square‐shaped (side length = 82 mm) RF‐transparent DC coils with three float traps (Figure 1B,C). Each balun has an inner/outer diameter of 5.8/17 mm and a length of 60 mm, with one end shorted and the other terminated with four capacitors (total capacitance ˜103 pF; Figure 1D,E). Figure 1G shows the assembled 16‐turn RF‐transparent DC coil.

Housings were 3D‐printed with a Form3 printer using Tough 2000 material. Thirty‐five‐μm‐thick copper foils were used as the conductors for the float trap. The DC coils were made of 0.51‐mm‐diameter 24‐AWG magnet copper wire. Every trap was tuned to 127.8 MHz and installed on the brackets, and magnet wire was threaded through the tubes. As a comparison, a conventional DC coil with the same dimensions and the same number of turns but without the sleeve float traps was also constructed (Figure 1F). The conventional and RF‐transparent DC coils were connected to a pair of twisted wires with toroid RF chokes.

To evaluate the response of the DC coils to the RF field, we measured the transmission coefficient (S_21_) of a pair of well‐decoupled double pick‐up probes when they were placed 2.5 cm above the DC coils. The probes were well decoupled without the presence of DC coils (with S_21_ < −70 dB) by carefully adjusting their overlapped area.

### 2.2 MRI experiments

All experiments were performed on a Philips 3T Elition scanner (Eindhoven, Netherlands) with a two‐channel body RF transmit coil.

To assess whether the proposed design could achieve the expected RF‐transparent performance, we measured the SNR and B_1_^+^ on a 19‐cm‐diameter FBIRN phantom^16^ without and with the RF‐transparent DC coil. A commercial receive coil from Philips (inner dimension ˜6 × 5 cm^2^) was placed on top of the phantom for signal reception,^17^ while the body coil was used for transmission. The impact of DC coils depends on its position relative to the RF coil, so two scenarios were evaluated. In the first scenario, the DC coil was placed stacked on the receive coil, providing a strong chance of strong coupling; in the second scenario, the DC coil was rotated to the right side of the phantom without any overlap with the RF coil, representing a strong chance of weak coupling. For comparison, we repeated the same experiments using the conventional DC coil.

SNR maps were calculated from gradient‐echo images as *SI*/*std*(noise) × 0.655, where *SI* is the signal intensity, and *std*(noise) is the standard deviation of the noise‐only maps.^18^ Gradient‐echo images were acquired on the central slice of the phantom using the following parameters: repetition time = 50 ms, echo time (TE) = 4.0 ms, field of view = 200 × 200 mm^2^, matrix = 240 × 240, slice thickness = 1.5 mm, flip angle = 20°, and bandwidth = 289.6 Hz/pixel. The noise‐only maps were acquired with the same parameters, but with the RF power turned off.

Multislice axial B_1_^+^ maps were measured using the dual repetition time method^19^ and the dual refocusing echo acquisition mode (DREAM) method,^20^ with the following parameters: field of view FOV = 200 × 200 mm^2^, matrix = 224 × 224, slice thickness = 3 mm. Both B_1_^+^ mapping methods were provided by Philips.

To demonstrate the high ∆B_0_/Amp ability of the RF‐transparent DC coils in B_0_ shimming, we performed two shimming experiments on a 350 × 250 × 200 mm^3^ cuboid tap water phantom containing a metallic hip implant with a Cobalt Chromium head and a Titanium stem (Zimmer Biomet Inc., Warsaw, IN, USA). The implant's ball was positioned 6 cm from the right wall of the phantom and centrally in vertical and longitudinal directions (Figure 5B). Two RF‐transparent coils were positioned head‐to‐head outside the phantom such that the coils' central gap aligned with the center of the implant's ball, which was 7 cm from it. A 32‐channel Philips body array was used for reception, whereas the body coil was used for transmission.^20^ The DC coils were driven with a three‐channel constant‐current DC power supply (Rigol DP832E; Rigol Inc., China).

Dual‐TE, gradient echo–based B_0_ mapping with the following parameters was performed to demonstrate the improvement in B_0_ homogeneity with local shimming: repetition time = 50 ms, TE/ΔTE =1.71/0.5 ms, field of view = 300 × 300 mm^2^, voxel size = 3 × 3 × 3 mm^3^, flip angle = 20°, and bandwidth = 1984.1 Hz/pixel. Field maps were acquired under two conditions: with the scanner's second‐order spherical harmonic shimming (SH2), which represented the best case shimming possible in the scanner, and with both the SH2 shim and local coil shim (SH2 + LC). A 4 × 4 cm^2^ region of interest adjacent to the implant was targeted for shimming, as shown in Figure 5G,H. The location of the region of interest next to the head of the implant represented an extreme case in which the off‐resonance exceeded −1000 Hz. Before imaging in the presence of the implant, unit field maps were first acquired with 1 Amp in each coil in the absence of the implant, but with the same coil setup. For shimming in the presence of the implant, an initial target field map was acquired without any current in DC coils. Together, they determined the optimal current in each DC coil to minimize the B_0_ inhomogeneity in the targeted region of interest.

To demonstrate the efficacy of shimming in alleviating distortions around the implant, a 33/42‐mm inner/outer diameter polycarbonate cylinder containing seven smaller 11‐mm outer‐diameter cylinders was introduced in the center of the phantom. Both single‐shot and multishot echo‐planar imaging scans were performed. Additionally, a multislice turbo spin‐echo image was also acquired to serve as the reference for assessing the distortions in the echo‐planar images. The scan parameters are detailed in the caption of Figure 5.

## 3 RESULTS

### 3.1 Bench test results

**FIGURE 2:**
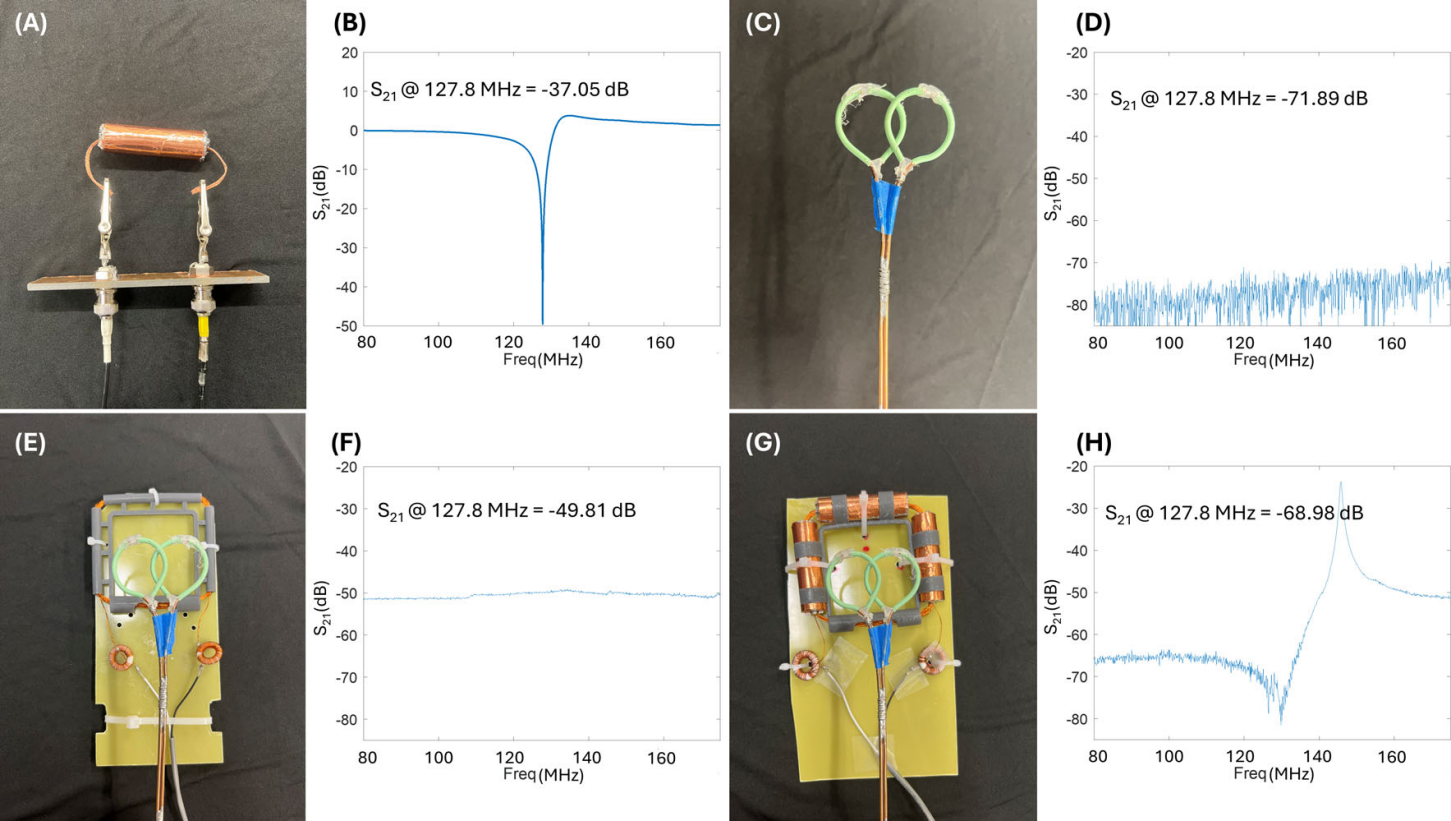
Bench test results demonstrating the blocking capabilities of the normal direct‐current (DC) coil and transparent DC coil. The probes were made of semirigid cables, and each probe had a diameter of 2.8 cm. The double pick‐up probes were placed 2.5 cm above the DC coils when the S_21_ plots were recorded. (A) Setup for measuring S_21_ of a single balun. (B) S_21_ measurement for a balun, showing effective blocking at 127.8 MHz with a value of −37.05 dB. (C) Double probes used for the S_21_ measurements. (D) Baseline S_21_ measurement at 127.8 MHz with the probes positioned 25 mm apart, showing a value of −71.89 dB. (E) Test setup for the normal DC coil configuration. (F) S_21_ measurement for the normal DC coil at 127.8 MHz, showing a value of −49.81 dB. (G) Test setup for the transparent DC coil configuration. (H) S_21_ measurement for the transparent DC coil at 127.8 MHz, showing a value of −68.98 dB.

Figure 2A,B shows that each float trap can reduce the RF signal to −37 dB, which is less than 0.1% residual. Figure C–H shows the measured S_21_ plots of double probes. The S_21_ parameter indicates how the DC coil responds to the RF field, with lower S_21_ values signifying better RF transparency. For the conventional DC coil, the S_21_ increased from −71.89 dB (baseline, Figure 2D) to −49.81 dB (Figure 2F) at 127.8 MHz. In contrast, the proposed RF‐transparent DC coil exhibited an S_21_ value of −68.98 dB (Figure 2H) at 127.8 MHz. This result indicates that the RF‐transparent DC coil introduces significantly less interference with the RF field and should, therefore, have a much lower impact on the performance of the RF coil.

### 3.2 SNR and B_1_ ^+^ results

**FIGURE 3:**
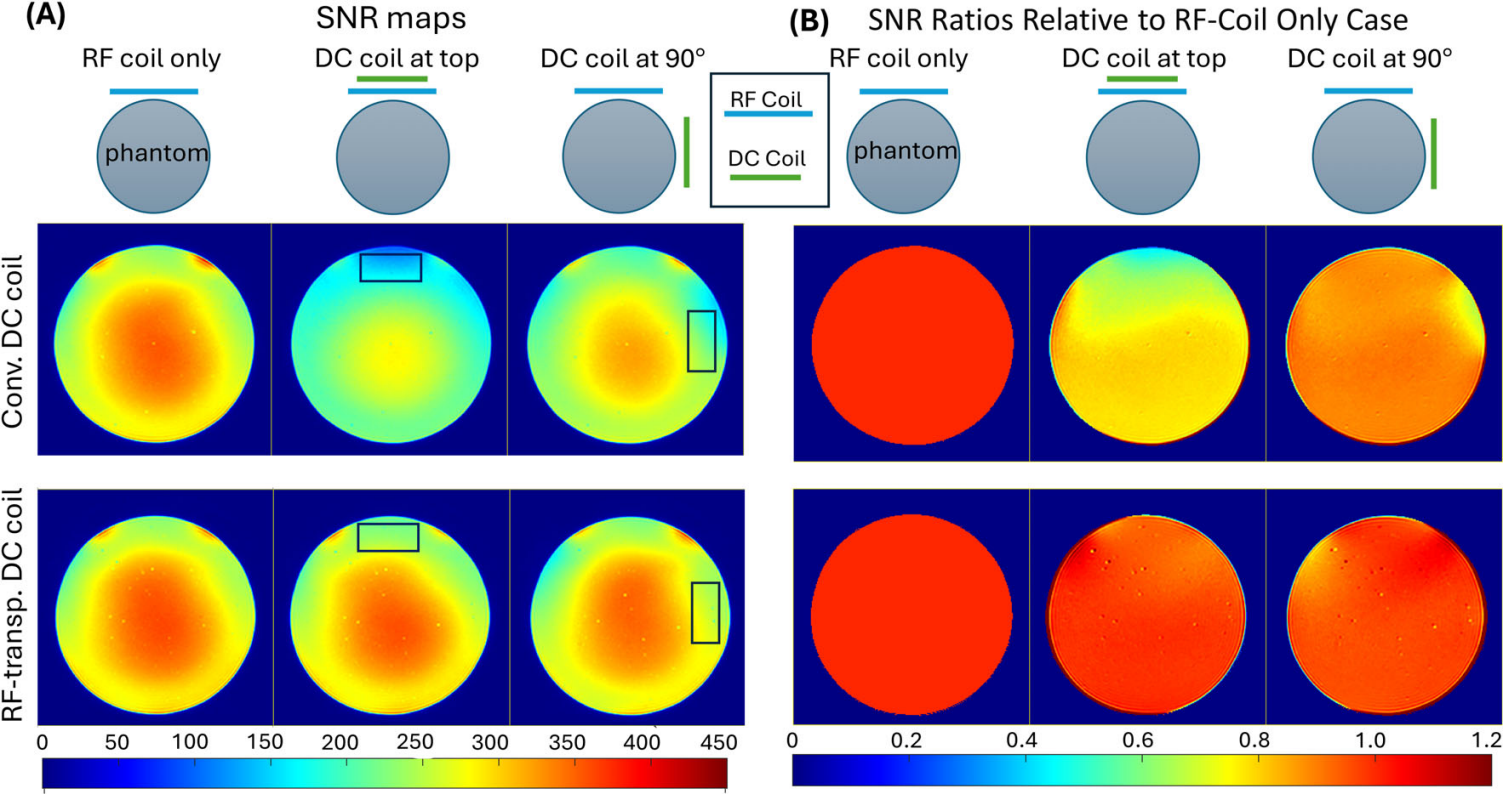
Signal‐to‐noise ratio (SNR) maps and SNRs for conventional direct‐current (DC) coil and radiofrequency (RF)–transparent DC coil. (A) SNR maps showing the SNR with different coil configurations: RF coil only, DC coil at the top, and DC coil at 90°. The top row displays the results for the conventional DC coil, whereas the bottom row shows the results for the RF‐transparent DC coil. The color scale represents the SNR values, with blue indicating low SNR and red indicating high SNR. (B) SNR ratios relative to the RF coil–only case for the same configurations. The left column shows the baseline SNR ratio with only the RF coil; the middle column shows the SNR ratio with the DC coil at the top; and the right column shows the SNR ratio with the DC coil at 90°. The top row represents the conventional DC coil, and the bottom row represents the RF‐transparent DC coil. The color scale represents the SNR ratio, with blue indicating a lower SNR ratio and red indicating a higher SNR ratio.

Figure 3A shows the measured SNR maps using the Philips single‐channel coil: without any DC coils, with the conventional DC coil, and with the RF‐transparent DC coil. Figure 3B presents the SNR ratios compared with the RF coil only. When directly stacked on the receive coil, the conventional DC coil results in a severe SNR drop (overall/local SNR decreases of 25.1%/40.7%). This SNR drop is alleviated when the DC coil is rotated to the side of the phantom, with overall/local SNR decreases of 11.9%/16.9%. In the latter configuration, the partially orthogonal orientation of the DC coil relative to the RF coil and their relatively large distance result in less mutual coupling and, thereby, less impact on the RF coil's receive performance. However, it is important to note that the SNR drop remains notable even in this scenario.

In contrast, the RF‐transparent DC coil does not affect the SNR when placed on the side of the phantom, with a negligible SNR decrease compared with RF coil only (overall/local difference < 2%). Even when directly stacked on the RF coil, the overall/local SNR decreases are only 2.8% and 4.9%.

**FIGURE 4:**
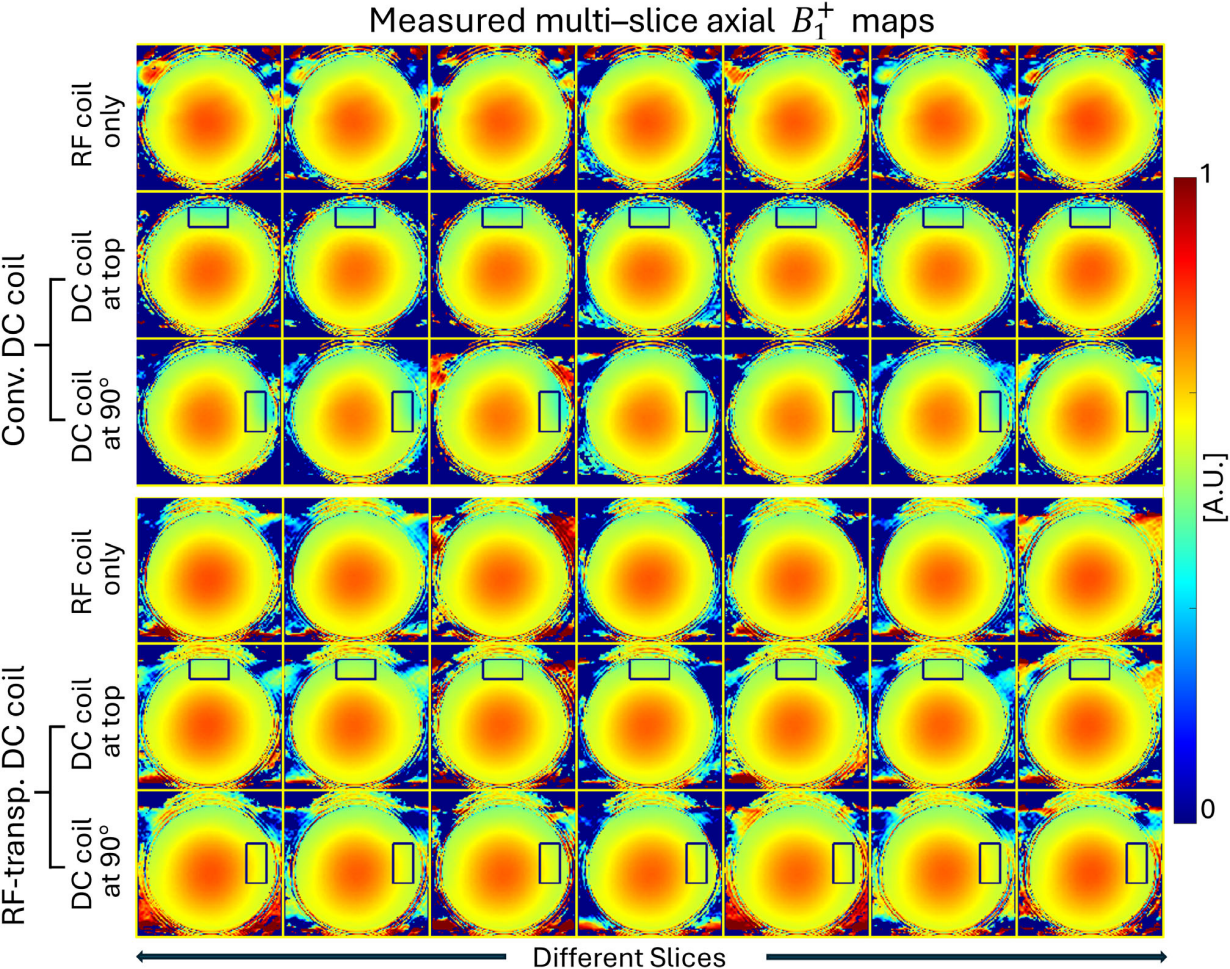
Measured multislice axial B_1_^+^ maps. Comparison of B_1_^+^ maps across different slices for various coil configurations. The top three rows display the B_1_^+^ maps for the conventional direct‐current (DC) coil with three different configurations: radiofrequency (RF) coil only, DC coil at top, and DC coil at 90°. The bottom three rows show the B_1_^+^ maps for the RF‐transparent DC coil under the same three configurations. The color scale represents the B_1_^+^ field distribution, with blue indicating low B_1_^+^ values and red indicating high B_1_^+^ values. The rectangles highlight regions where deviations in the B_1_^+^ field were observed due to the presence of the coils.

Figure 4 shows the multislice axial B_1_^+^ maps using the dual repetition time and DREAM methods. Surprisingly, the conventional DC coil results in an approximate 13.1% local B_1_^+^ drop, even when it is more than 20 cm away from the inner side of the scanner bore. In contrast, the RF‐transparent DC coil has almost no effect on the B_1_^+^, with only an approximate 1.2% difference. These results indicate that, without careful attention, the DC coil can cause notable B_1_^+^ changes in the body coil, raising safety concerns as it could also alter the associated electrical field and local specific absorption rate distribution. The overall SNR was averaged over the whole image area, whereas the local SNR and B_1_^+^ were averaged over rectangular areas of 5.1 × 2.2 cm^2^ and 5.64 × 2.8 cm^2^, respectively (dark blue boxes in Figures 3 and 4).

Both the SNR and B_1_^+^ results are consistent with the bench test results and further prove that the proposed design is almost transparent to both the RF transmit field and the RF receive field. These results also confirm that RF transparency is crucial, as it directly affects the SNR and specific absorption rate.

### 3.3 B_0_ shimming results near a metal hip implant

**FIGURE 5:**
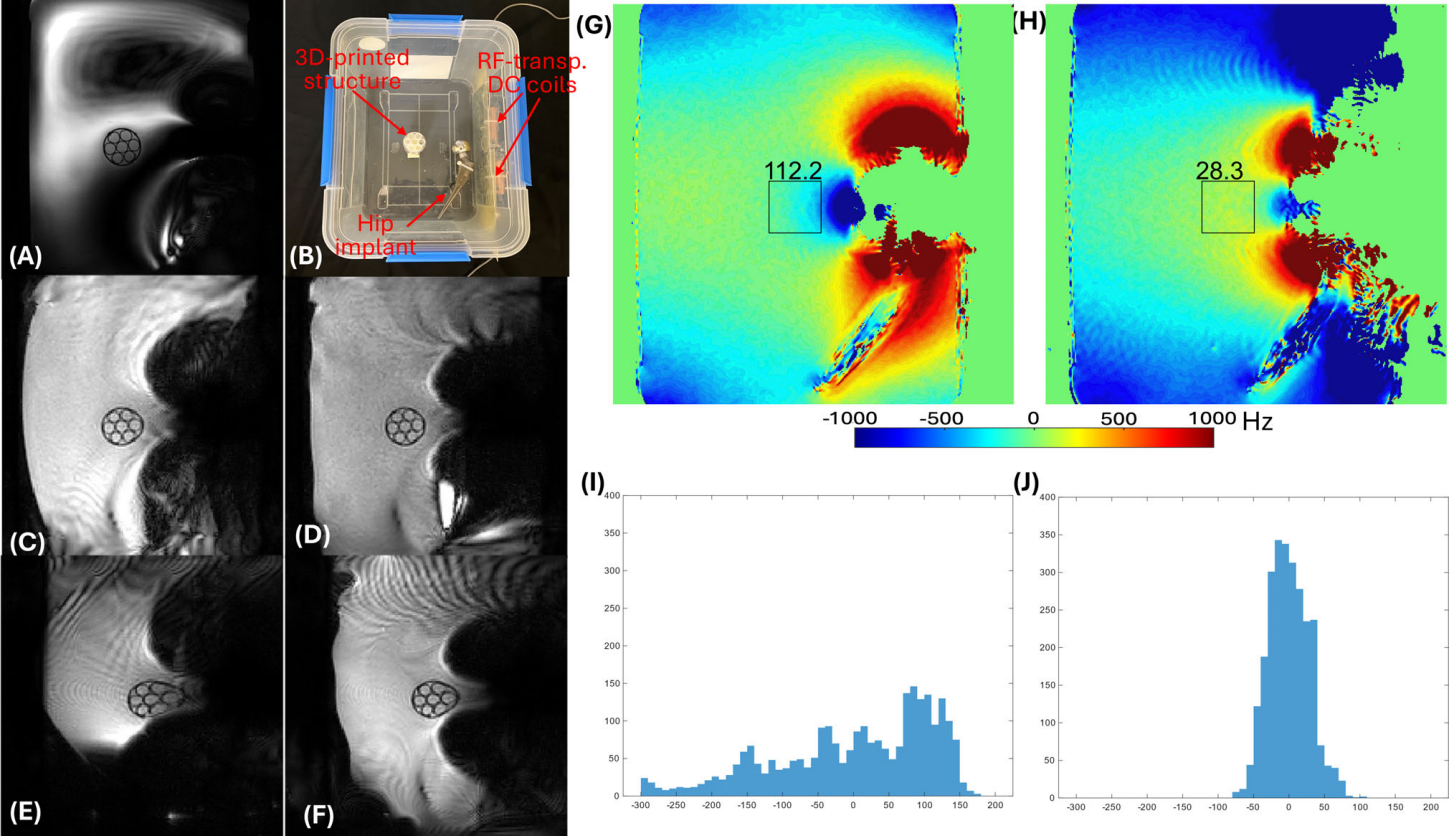
Shimming evaluation with hip implant phantom. (A) Reference turbo spin‐echo (TSE) scan. (B) Experimental setup with the hip implant phantom, three‐dimensional (3D)–printed structure, and radiofrequency (RF)–transparent direct‐current (DC) coils. (C–F) Multishot and single‐shot echo‐planar images (EPI) without shimming (C,E) and with optimal shim currents (D,F), demonstrating improved image quality and reduced distortion with shimming. TSE scan was performed with the following parameters: field of view (FOV) = 300 × 300 mm^2^, repetition time (TR)/echo time (TE) = 2500/251.6 ms, matrix = 320 × 320, TSE factor = 120, flip angle (FA) = 90°, slice thickness = 1 mm, and bandwidth (BW) = 947 Hz/pixel. Single‐shot EPI was performed with the following parameters: FOV = 300 × 300 mm^2^, slice thickness = 2.0 mm, and matrix = 160 × 160, TR/TE = 1445 /48 ms, FA = 90°, EPI factor = 75, BW (phase/frequency encode direction) = 11.0/1314.4 Hz/pixel. Multishot EPI was performed with the same FOV, slice thickness, FA, and matrix size as previously and with a TR/TE = 350/11 ms, EPI factor = 13, and BW (phase/frequency encode direction) = 55.4/1309.6 Hz/pixel. Shimming reduced distortions and signal pileups and improved image quality significantly in both single‐shot and multishot EPI. (G) B_0_ field map before shimming highlighting severe inhomogeneity around the implant. (H) B_0_ field map after shimming, showing a significant reduction in field inhomogeneity. (I,J) Histograms of the B_0_ field distribution before and after shimming, respectively, indicating a more uniform field after shimming with the transparent DC coil.

Figure 5G,H shows the results of the B_0_ shimming experiments. The shim fit estimated the need for 2.5 A in Coil #1 and 2 A in Coil #2 (near the entrance of the patient bed to the scanner bore). With these currents in the shim coils, the standard deviation of the field in the region of interest decreased from 111.2 Hz with SH2 to 28.3 Hz with SH2 + LC, demonstrating significant improvement in the field profile with just two RF‐transparent DC coils. The image consists of 15 slices in total, and the standard deviation drop was tested on the middle (eighth) slice.

Figure 5E,F shows multishot and single‐shot echo‐planar images with SH2 shimming, whereas Figure 5C,D shows those with SH2 + LC shimming. A significant reduction in the distortion of the cylindrical structure is achieved with SH2 + LC shimming, which agrees with the improvement in the field homogeneity (Figure 5G–J). A turbo spin‐echo image in Figure 5A gives a low‐distortion reference for the cylindrical structure.

## 4 DISCUSSION AND CONCLUSION

We have introduced and validated the concept of an RF‐transparent local DC coil for B_0_ shimming in MRI. Our innovative design, using float traps to achieve high RF impedance on every turn of the DC coils, has demonstrated minimal impact on RF performance, thus enabling more flexible placement and multiturn configurations of the DC coils. Bench tests exhibited significant improvements in RF transparency compared with conventional DC coils, as evidenced by the workbench results. MRI experiments confirmed that the RF‐transparent DC coil has negligible RF interference and does not impair the SNR or B_1_^+^. It is important to note that non‐RF‐transparent DC coils affect the electric field, influencing local specific absorption rate distributions and potentially raising safety concerns.

The float trap's self‐shielding capability ensures it does not couple with the local or body coil. In this work, we used a continuous piece of copper foil for the outer conductor of the float trap. Segmented copper foils could be used instead to reduce eddy currents and prevent potential heating from the gradient. We used three float traps; however, the number of float traps may be reduced while maintaining the same level of transparency to the RF field, as demonstrated in Figure S1.

Some limitations of this work should be mentioned. Float baluns result in an increased footprint and added complexity in the fabrication of the DC coil. As a proof of concept, we did not optimize the individual size and number of float baluns. Based on bench tests demonstrating a common mode attenuation ability of −37 dB, the size could be further reduced without compromising the RF‐transparent feature. Additionally, the float feature of the trap allows closely placed wires from different DC coils to share the same set of float traps, thereby reducing the overall footprint and bulkiness. Although wiring the RF‐transparent DC coil is more laborious than wiring a standard DC coil, it is manageable, as each coil requires only a few float traps to achieve sufficient RF transparency. Further studies are needed to explore new fabrication processes to simplify the production of such DC coils, such as split balun design and multi‐material three‐dimensional printing technology.

Shimming around an implant demands high shim efficiency, which is difficult to achieve with traditional shim coil designs. For example, the field produced by the 2.5A, 16‐turn coil used here would require approximately 40A in a single‐turn combined RF‐DC coil, which is practically difficult. Although we used only two RF‐transparent coils with a fixed spatial configuration for an initial demonstration here, adding more coils can provide the degrees of freedom needed to reduce inhomogeneities further and improve imaging around metallic implants in general.

This innovative approach presents new opportunities for designing and implementing shimming coils in MRI, offering greater flexibility in coil architecture and proximity to the imaging area. The capability to use many‐turn coils and irregular shapes allows for improved shimming performance. A notable advantage of this work is that such coils can seamlessly integrate with commercial RF coils due to their RF field transparency. This underscores the significant potential of our design alongside existing commercial RF coils, particularly for clinical applications. Our RF‐transparent DC coils can be adopted and implemented in clinical MRI without requiring hardware modifications to existing RF coils.

## CONFLICT OF INTEREST

Nothing to report.

## Supporting information


**Figure S1.** Bench test setups and results demonstrating the S21 measurements for various radiofrequency (RF) transparent direct‐current (DC) coil configurations. The double pick‐up probes were placed 2.5 cm above the DC coils for the S21 measurement. (A) Double probes used for the S21 measurements. (B) Baseline S21 measurement at 128 MHz, showing a value of −79.57 dB. (C,D) Test setup and measured S21 for the RF‐transparent DC coil with a single float balun positioned on top. (E,F) Test setup and measured S21 for the RF‐transparent DC coil with three float baluns. (G,H) Test setup and measured S21 for the RF‐transparent DC coil with a single float balun positioned on left. Based on the bench test results, the number of float traps may be reduced while maintaining the same level of transparency to the RF field. However, the definitive assessment of RF transparency should still be determined by the SNR and B_1_^+^ results.
