# Supplementary material for: Radiofrequency‐transparent local B0 shimming coils using float traps
Source: Magn Reson Med. 2024 Nov 4;93(4):1833–41. doi: 10.1002/mrm.30361 (PMC11782720; doi:10.1002/mrm.30361)
Supplement: Supplementary file 1 — Figure S1. Bench test setups and results demonstrating the S21 measurements for various radiofrequency (RF) transparent direct‐current (DC) coil configurations. The double pick‐up probes were placed 2.5 cm above the DC coils for the S21 measurement. (A) Double probes used for the S21 measurements. (B) Baseline S21 measurement at 128 MHz, showing a value of −79.57 dB. (C,D) Test setup and measured S21 for the RF‐transparent DC coil with a single float balun positioned on top. (E,F) Test setup and measured S21 for the RF‐transparent DC coil with three float baluns. (G,H) Test setup and measured S21 for the RF‐transparent DC coil with a single float balun positioned on left. Based on the bench test results, the number of float traps may be reduced while maintaining the same level of transparency to the RF field. However, the definitive assessment of RF transparency should still be determined by the SNR and B1 + results. [file MRM-93-1833-s001.pdf]

Supplementary Materials

The Supplementary Information includes 1 Supporting Figure.

RF-Transparent Local B_0 Shimming Coils using Float Traps

Changzhe Liu [a, b], Hao Liang [b, c], Ming Lu [b, c], John C. Gore [b, c], Saikat Sengupta [b, c] and Xinqiang Yan [a, b, c]

a. Department of Electrical and Computer Engineering,
Vanderbilt University, Nashville, TN, 37232, USA

b. Vanderbilt University Institute of Imaging Science,
Vanderbilt University Medical Center, Nashville, TN, 37232, USA

c. Department of Radiology and Radiological Sciences,
Vanderbilt University Medical Center, Nashville, TN, 37232, USA

Corresponding Author:

Xinqiang Yan, Ph.D.

Vanderbilt University Institute of Imaging Science

1161 21st Avenue South
Medical Center North, D-2205
Nashville, TN 37232-2310

Phone No: 1(615) 5253989

Email: xinqiang.yan@vumc.org

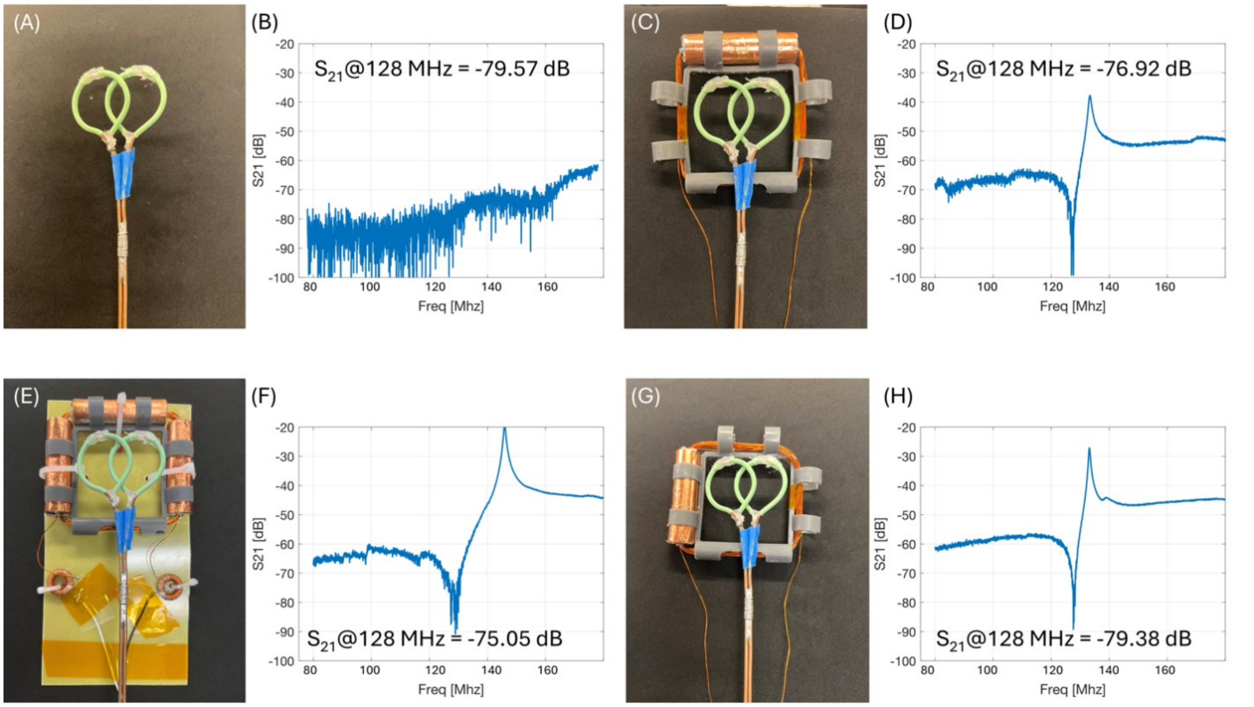

Figure S1 Bench Test Setups and Results Demonstrating the S_{21} Measurements for Various RF-Transparent DC Coil Configurations. The double pick-up probes were placed 2.5 cm above the DC coils when the S_{21} measurement. From A to H: (A) Double probes used for the S_{21} measurements. (B) Baseline S_{21} measurement at 128 MHz, showing a value of -79.57 dB. (C and D) Test setup and measured S_{21} for the RF-transparent DC coil with a single float balun positioned on top. (E and F) Test setup and measured S_{21} for the RF-transparent DC coil with three float baluns. (G and H) Test setup and measured S_{21} for the RF-transparent DC coil with a single float balun positioned on left. Based on the bench test results, the number of float traps may be reduced while maintaining the same level of transparency to the RF field. However, the definitive assessment of RF transparency should still be determined by the SNR and B_1^+ results.
